# Analysis of disparate factors affecting cognitive function among populations with different educational levels: a large-scale longitudinal study

**DOI:** 10.3389/fpsyg.2026.1564721

**Published:** 2026-03-19

**Authors:** Dezhen Dai, Bingbing Xiang, Yunke Dai, Pingliang Yang, Na Zhu, Shun Wang

**Affiliations:** 1 Outpatient Department, Clinical Medical College and The First Affiliated Hospital of Chengdu Medical College, Chengdu, Sichuan, China; 2 Department of Anesthesiology, West China Hospital, Sichuan University, Chengdu, China; 3 Department of Anesthesiology, Clinical Medical College and The First Affiliated Hospital of Chengdu Medical College, Chengdu, Sichuan, China

**Keywords:** China Health and Retirement Longitudinal Study (CHARLS), cognitive function, educational attainment, longitudinal study, population aging

## Abstract

**Objective:**

To explore the influencing factors and patterns of cognitive function among populations with different educational levels.

**Methods:**

Using data from the China Health and Retirement Longitudinal Study (2015–2020), we analyzed 29,620 subjects aged 45–85 years who completed cognitive function assessments. Participants were stratified by educational level: illiterate (*n* = 7,670), primary school (*n* = 7,897), junior high school (*n* = 8,904), and high school and above (*n* = 5,149). Mixed-effects models were used to analyze cognitive function determinants across educational groups, with sensitivity analyses performed to verify result robustness.

**Results:**

Cognitive function scores demonstrated a significant educational gradient, with the highest scores in the high-education group [20.0 (18.0–23.0)] and lowest in the illiterate group [16.0 (13.0–18.0)]. Age negatively correlated with cognitive function, with stronger effects in higher education groups (illiterate: *β* = −0.665; high-education: *β* = −1.033, *p* < 0.001). Gender effects varied by education level: males showed cognitive advantages in the illiterate group (*β* = 0.716, *p* < 0.001), but disadvantages in the high-education group (*β* = −0.739, *p* < 0.001). Internet use demonstrated enhanced protective effects with increasing education (illiterate: *β* = 0.254; high-education: *β* = 0.411, *p* < 0.001). Urban residence benefits strengthened with education level (illiterate: *β* = 0.188; high-education: *β* = 0.439, *p* < 0.001). Memory-related diseases showed the most significant impact in the high-education group (*β* = −2.325, *p* < 0.05).

**Conclusion:**

Educational level appears to act not only as an independent correlate of cognitive function but also as a potential modifier of its associations with gender, Internet use, residential environment, and chronic disease burden. These observations suggest educational background warrants consideration when designing cognitive health strategies for older adults.

## Highlights

- Gender effects on cognition vary by education
- Internet’s cognitive protection increases with educational level
- Age-related cognitive decline intensifies at higher educational levels
- Memory-related diseases show the most significant impact in high-education groups

## Introduction

Cognitive impairment has emerged as a major public health challenge amid global population aging, significantly impacting elderly quality of life and healthcare systems (He et al., 2024; Luo et al., 2024). This condition not only increases mortality and disability risk but also creates substantial socioeconomic burden (Tahami Monfared et al., 2022). Current estimates indicate approximately 132 million individuals worldwide have mild cognitive impairment, with projections reaching 152 million by 2050, demonstrating marked demographic and geographic variation (GBD 2019 Dementia Forecasting Collaborators, 2022).

Educational attainment, a key cognitive reserve indicator, fundamentally influences cognitive trajectory (Bratsberg et al., 2024; Singh-Manoux et al., 2011). The cognitive reserve hypothesis posits that higher education enhances neural plasticity and network optimization, potentially moderating cognitive decline (Barba et al., 2021). Education’s impact likely extends beyond direct effects, with emerging evidence suggesting its role as an effect modifier (Jia et al., 2022). Studies indicate education may indirectly affect cognition by shaping health behaviors, social engagement patterns, and self-management capabilities (Jia et al., 2022; Nguyen et al., 2013).

Contemporary societal changes have introduced new cognitive function determinants. Digital technology adoption, particularly Internet use, may serve as a cognitive protective factor (Ihle et al., 2020; Amland et al., 2024). Evolving modes of social participation provide novel cognitive engagement opportunities (Deng et al., 2019; Siette et al., 2020). Additionally, changing chronic disease patterns, especially the rising prevalence of metabolic disorders, present new considerations in cognitive health (Sanchez-Orti et al., 2022; Maksyutynska et al., 2024).

Prior investigations have largely treated education as a confounder rather than exploring its potential effect modification (He et al., 2024; Ghahremani et al., 2024). Most studies employed cross-sectional designs, limiting understanding of temporal relationships (Clare et al., 2017; Sedgwick, 2015). The China Health and Retirement Longitudinal Study (CHARLS), a nationwide prospective cohort study, offers an opportunity to address these limitations (Zhu et al., 2024). Using probability-proportional-to-size sampling, CHARLS collected comprehensive data on cognitive function and its determinants across educational strata.

The present study examined CHARLS data from 2015 to 2020 to address three key questions: (1) whether cognitive function determinants differ systematically across educational levels, (2) how these relationships evolve over time, and (3) the extent and nature of effect modification by educational attainment. This investigation advances current knowledge by analyzing education’s moderating role using longitudinal data, examining temporal trends in cognitive risk factors, and employing multiple sensitivity analyses to ensure robust findings. Results may inform the development of education-specific cognitive intervention strategies.

## Methods

### Research design and data source

We conducted a prospective cohort study using data from the CHARLS collected in 2015, 2018, and 2020. CHARLS employed multi-stage stratified probability sampling, covering 150 counties across 28 Chinese provinces, targeting residents aged ≥45 years (Zhao et al., 2014). This study was conducted in accordance with the Declaration of Helsinki and was approved by the Biomedical Ethics Review Committee of Peking University (IRB00001052-11015), and all participants provided written informed consent.

### Study population

Inclusion criteria comprised: (1) age 45–85 years; (2) completion of cognitive function assessment; and (3) available educational attainment data. We excluded participants with missing key variables or outlier cognitive assessment scores. The final analytic sample included 14,236 participants with complete data across all three waves (29,620 observations). Participants were stratified by educational level into four groups: illiterate (*n* = 7,670), primary school (*n* = 7,897), junior high school (*n* = 8,904), and high school and above (*n* = 5,149).

### Variable measurement

#### Primary outcome variable

The cognitive function total score (range: 0–31) comprised four domains: Memory (0–20 points): immediate word recall (10 words, 0–10 points) and delayed word recall (0–10 points); Orientation (0–5 points): assessment of temporal orientation (year, month, day, week, time); Calculation (0–5 points): serial subtraction by 7 s; Visuospatial ability (0–1 point): pentagon copying task

#### Independent variables

Demographic characteristics: age (continuous), sex (male/female), marital status (married/unmarried); Lifestyle factors: smoking and alcohol consumption (current or former/never), sleep duration (<8 h, ≥8 h), napping (yes/no), Internet use (yes/no), social activity participation (yes/no); Environmental factors: residence (urban/rural), life satisfaction (satisfied/unsatisfied); Health status: chronic conditions (14 common diseases), physical pain (yes/no).

#### Statistical analysis

The dataset comprised predominantly binary and categorical variables; age and cognitive function total score were the only continuous measures. Normality of these variables was assessed using the Shapiro–Wilk test prior to analysis. Neither variable satisfied the normality criterion in any educational subgroup, and continuous variables were therefore summarized as median (interquartile range). Between-group differences were examined using Kruskal–Wallis tests with Bonferroni correction for post-hoc pairwise comparisons. Categorical variables were expressed as frequency (percentage) and compared using chi-square tests, with Fisher’s exact test applied where expected cell counts fell below five. Spearman correlation assessed relationships between cognitive function and potential predictors.

Multiple linear regression models were used to examine factors associated with cognitive function in each educational group, adjusting for demographic, lifestyle, environmental, and health-related variables. Standardized coefficients (*β*) were reported for all associations.

We constructed four sequential models: Model 1: demographic characteristics only; Model 2: Model 1 + lifestyle factors; Model 3: Model 2 + environmental factors; Model 4: Model 3 + health status factors. All models included year and individual-level random effects. Residual diagnostics including Q-Q plots and density curves were examined for all subgroup models, confirming that the normality assumption for model residuals was adequately satisfied. To formally test whether educational level functions as an effect modifier, we fitted a pooled mixed-effects model incorporating interaction terms between educational level and five key predictors (age, sex, internet use, urban residence, and memory-related disease). All Model 4 covariates were retained as main effects, with an identical random-effects structure. Interaction significance was assessed using likelihood ratio tests and Type III F-tests (Satterthwaite approximation).

We tested result robustness using two alternative educational stratification schemes: Binary classification: low (illiterate + primary school) versus high (junior high school + high school and above). Three-level classification: illiterate, medium (primary + junior high school), and high education. Statistical analyses were performed using R version 4.0.3, with two-sided *p* < 0.05 considered statistically significant.

## Results

### Baseline characteristics

**Figure 1:**
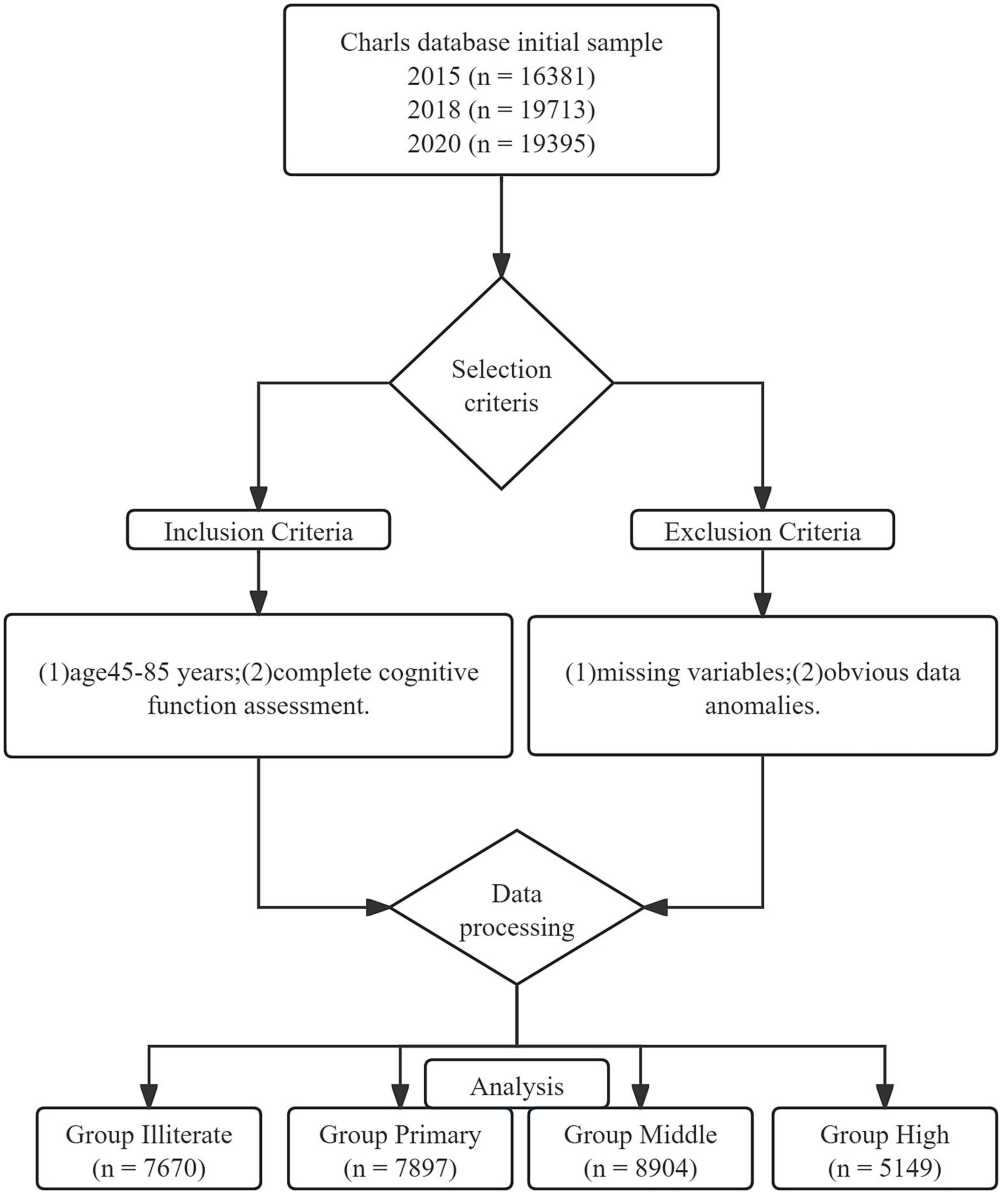
Flowchart of the sample selection process.

Based on CHARLS data from 2015–2020, we included 29,620 participants: 9,145 in 2015, 9,979 in 2018, and 10,496 in 2020. Participants were stratified by educational level into illiterate (*n* = 7,670), primary school (*n* = 7,897), junior high school (*n* = 8,904), and high school and above groups (*n* = 5,149) (Table 1; Figure 1).

**Table 1:** Characteristics of the study population from CHARLS.

Variable	Category	Overall (*N* = 29,620)	Group Illiterate (*N* = 7,670)	Group Primary (*N* = 7,897)	Group Middle (*N* = 8,904)	Group High (*N* = 5,149)	*p*
Life satisfaction (*n*, %)	Satisfied	27,206 (91.9%)	6,957 (90.7%)^c,d^	7,207 (91.3%)^c,d^	8,222 (92.3%)^a,b,d^	4,820 (93.6%)^a,b,c^	<0.001
	Dissatisfied	2,414 (8.1%)	713 (9.3%)^c,d^	690 (8.7%)^c,d^	682 (7.7%)^a,b,d^	329 (6.4%)^a,b,c^	
Gender (*n*, %)	Female	14,155 (47.8%)	4,396 (57.3%)^b,c,d^	3,726 (47.2%)^a,c,d^	3,936 (44.2%)^a,b,d^	2097 (40.7%)^a,b,c^	<0.001
	Male	15,465 (52.2%)	3,274 (42.7%)^b,c,d^	4,171 (52.8%)^a,c,d^	4,968 (55.8%)^a,b,d^	3,052 (59.3%)^a,b,c^	
Marital status (*n*, %)	Unmarried/divorced/widowed/separated	3,562 (12.0%)	965 (12.6%)	969 (12.3%)	1,035 (11.6%)	593 (11.5%)	0.151
	Married	26,058 (88.0%)	6,705 (87.4%)	6,928 (87.7%)	7,869 (88.4%)	4,556 (88.5%)	
Ever/current smoke (*n*, %)	No	16,555 (55.9%)	4,798 (62.6%)^b,c,d^	4,389 (55.6%)^a,c,d^	4,647 (52.2%)ab	2,721 (52.8%)ab	<0.001
	Yes	13,065 (44.1%)	2,872 (37.4%)^b,c,d^	3,508 (44.4%)^a,c,d^	4,257 (47.8%)ab	2,428 (47.2%)ab	
Ever/current alcohol (*n*, %)	No	17,525 (59.2%)	4,737 (61.8%)^b,c,d^	4,731 (59.9%)^a,c,d^	5,157 (57.9%)ab	2,900 (56.3%)ab	<0.001
	Yes	12,095 (40.8%)	2,933 (38.2%)^b,c,d^	3,166 (40.1%)^a,c,d^	3,747 (42.1%)^a,b^	2,249 (43.7%)^a,b^	
Age (years)		59.00 (52.00–66.00)	62.00 (54.00–68.00)^b,c,d^	59.00 (52.00–68.00)^a,c,d^	56.00 (52.00–63.00)^a,b,d^	58.00 (53.00–63.00)^a,b,c^	<0.001
Daily sleep time (h) (*n*, %)	< 8	22,072 (74.5%)	5,608 (73.1%)^c,d^	5,745 (72.7%)^c,d^	6,652 (74.7%)^a,b,d^	4,067 (79.0%)^a,b,c^	<0.001
	≥ 8	7,548 (25.5%)	2062 (26.9%)^c,d^	2,152 (27.3%)^c,d^	2,252 (25.3%)^a,b,d^	1,082 (21.0%)^a,b,c^	
Nap (*n*, %)	No	11,954 (40.4%)	3,433 (44.8%)^b,c,d^	3,310 (41.9%)^a,c,d^	3,469 (39.0%)^a,b,d^	1742 (33.8%)^a,b,c^	<0.001
	Yes	17,666 (59.6%)	4,237 (55.2%)^b,c,d^	4,587 (58.1%)^a,c,d^	5,435 (61.0%)^a,b,d^	3,407 (66.2%)^a,b,c^	
Internet (*n*, %)	No	22,510 (76.0%)	6,352 (82.8%)^b,c,d^	6,380 (80.8%)^a,c,d^	6,588 (74.0%)^a,b,d^	3,190 (62.0%)^a,b,c^	<0.001
	Yes	7,110 (24.0%)	1,318 (17.2%)^b,c,d^	1,517 (19.2%)^a,c,d^	2,316 (26.0%)^a,b,d^	1959 (38.0%)^a,b,c^	
Social activities (*n*, %)	No	12,854 (43.4%)	3,792 (49.4%)^b,c,d^	3,623 (45.9%)^a,c,d^	3,658 (41.1%)^a,b,d^	1781 (34.6%)^a,b,c^	<0.001
	Yes	16,766 (56.6%)	3,878 (50.6%)^b,c,d^	4,274 (54.1%)^a,c,d^	5,246 (58.9%)^a,b,d^	3,368 (65.4%)^a,b,c^	
Residential area (*n*, %)	Rural	19,327 (65.2%)	5,678 (74.0%)^b,c,d^	5,588 (70.8%)^a,c,d^	5,557 (62.4%)^a,b,d^	2,504 (48.6%)^a,b,c^	<0.001
	Urban	10,293 (34.8%)	1992 (26.0%)^b,c,d^	2,309 (29.2%)^a,c,d^	3,347 (37.6%)^a,b,d^	2,645 (51.4%)^a,b,c^	
Troubled with body pain (n, %)	No	16,922 (57.1%)	4,347 (56.7%)	4,448 (56.3%)	5,115 (57.4%)	3,012 (58.5%)	0.0711
	Yes	12,698 (42.9%)	3,323 (43.3%)	3,449 (43.7%)	3,789 (42.6%)	2,137 (41.5%)	
Co-morbidities (*n*, %)							
Hypertension	No	23,838 (80.5%)	6,008 (78.3%)^b,c,d^	6,368 (80.6%)ac	7,305 (82.0%)^a,b^	4,157 (80.7%)a	<0.001
	Yes	5,782 (19.5%)	1,662 (21.7%)^b,c,d^	1,529 (19.4%)ac	1,599 (18.0%)^a,b^	992 (19.3%)a	
Dyslipidaemia	No	26,516 (89.5%)	6,955 (90.7%)^c,d^	7,216 (91.4%)^c,d^	7,961 (89.4%)^a,b,d^	4,384 (85.1%)^a,b,c^	<0.001
	Yes	3,104 (10.5%)	715 (9.3%)^c,d^	681 (8.6%)^c,d^	943 (10.6%)^a,b,d^	765 (14.9%)^a,b,c^	
Hyperglycaemia	No	27,966 (94.4%)	7,252 (94.6%)^d^	7,470 (94.6%)^d^	8,432 (94.7%)^d^	4,812 (93.5%)^a,b,c^	0.0112
	Yes	1,654 (5.6%)	418 (5.4%)^d^	427 (5.4%)^d^	472 (5.3%)^d^	337 (6.5%)^a,b,c^	
Cancer	No	29,371 (99.2%)	7,606 (99.2%)	7,808 (98.9%)^c^	8,849 (99.4%)^b^	5,108 (99.2%)	0.004
	Yes	249 (0.8%)	64 (0.8%)	89 (1.1%)^c^	55 (0.6%)^b^	41 (0.8%)	
Chronic lung disease	No	27,406 (92.5%)	6,993 (91.2%)^c,d^	7,206 (91.2%)^c,d^	8,365 (93.9%)^a,b^	4,842 (94.0%)^a,b^	<0.001
	Yes	2,214 (7.5%)	677 (8.8%)^c,d^	691 (8.8%)^c,d^	539 (6.1%)^a,b^	307 (6.0%)^a,b^	
Liver disease	No	28,676 (96.8%)	7,438 (97.0%)	7,658 (97.0%)	8,619 (96.8%)	4,961 (96.3%)	0.178
	Yes	944 (3.2%)	232 (3.0%)	239 (3.0%)	285 (3.2%)	188 (3.7%)	
Heart disease	No	26,682 (90.1%)	6,906 (90.0%)^c,d^	7,091 (89.8%)c	8,103 (91.0%)^a,b,d^	4,582 (89.0%)^a,c^	0.001
	Yes	2,938 (9.9%)	764 (10.0%)^c,d^	806 (10.2%)c	801 (9.0%)^a,b,d^	567 (11.0%)ac	
Stroke	No	29,068 (98.1%)	7,511 (97.9%)	7,745 (98.1%)	8,758 (98.4%)	5,054 (98.2%)	0.217
	Yes	552 (1.9%)	159 (2.1%)	152 (1.9%)	146 (1.6%)	95 (1.8%)	
Kidney disease	No	28,172 (95.1%)	7,258 (94.6%)^c^	7,511 (95.1%)	8,515 (95.6%)^a^	4,888 (94.9%)	0.0246
	Yes	1,448 (4.9%)	412 (5.4%)^c^	386 (4.9%)	389 (4.4%)^a^	261 (5.1%)	
Digestive system disease	No	24,380 (82.3%)	6,110 (79.7%)^c,d^	6,375 (80.7%)^c,d^	7,493 (84.2%)^a,b,d^	4,402 (85.5%)^a,b,c^	<0.001
	Yes	5,240 (17.7%)	1,560 (20.3%)^c,d^	1,522 (19.3%)^c,d^	1,411 (15.8%)^a,b,d^	747 (14.5%)^a,b,c^	<0.001
Emotional / mental disorders	No	29,419 (99.3%)	7,608 (99.2%)	7,840 (99.3%)	8,848 (99.4%)	5,123 (99.5%)	0.190
	Yes	201 (0.7%)	62 (0.8%)	57 (0.7%)	56 (0.6%)	26 (0.5%)	
Memory-related disease	No	29,396 (99.2%)	7,606 (99.2%)	7,842 (99.3%)	8,848 (99.4%)	5,100 (99.0%)	0.136
	Yes	224 (0.8%)	64 (0.8%)	55 (0.7%)	56 (0.6%)	49 (1.0%)	
Arthritis or rheumatism	No	22,707 (76.7%)	5,412 (70.6%)^b,c,d^	5,933 (75.1%)^a,c,d^	7,045 (79.1%)^a,b,d^	4,317 (83.8%)^a,b,c^	<0.001
	Yes	6,913 (23.3%)	2,258 (29.4%)^b,c,d^	1964 (24.9%)^a,c,d^	1859 (20.9%)^a,b,d^	832 (16.2%)^a,b,c^	
Asthma	No	28,747 (97.1%)	7,406 (96.6%)^c,d^	7,591 (96.1%)^c,d^	8,698 (97.7%)^a,b^	5,052 (98.1%)^a,b^	<0.001
	Yes	873 (2.9%)	264 (3.4%)^c,d^	306 (3.9%)^c,d^	206 (2.3%)^a,b^	97 (1.9%)^a,b^	
Cognitive function score							
Total cognitive Scores		18.00 (15.00–21.00)	16.00 (13.00–18.00)^b,c,d^	18.00 (15.00–20.00)^a,c,d^	19.00 (16.00–21.00)^a,b,d^	20.00 (18.00–23.00)^a,b,c^	<0.001
Memory		9.00 (7.00–11.00)	8.00 (6.00–10.00)^b,c,d^	9.00 (7.00–11.00)^a,c,d^	10.00 (8.00–12.00)^a,b,d^	11.00 (9.00–13.00)^a,b,c^	<0.001
Orientation	1	393 (1.3%)	246 (3.2%)^b,c,d^	87 (1.1%)^a,c,d^	48 (0.5%)^a,b,d^	12 (0.2%)^a,b,c^	<0.001
	2	1,396 (4.7%)	706 (9.2%)^b,c,d^	337 (4.3%)^a,c,d^	283 (3.2%)^a,b,d^	70 (1.4%)^a,b,c^	
	3	4,332 (14.6%)	1,631 (21.3%)^b,c,d^	1,206 (15.3%)^a,c,d^	1,113 (12.5%)^a,b,d^	382 (7.4%)^a,b,c^	
	4	9,343 (31.5%)	2,530 (33.0%)^b,c,d^	2,638 (33.4%)^a,c,d^	2,819 (31.7%)^a,b,d^	1,356 (26.3%)^a,b,c^	
	5	14,156 (47.8%)	2,557 (33.3%)^b,c,d^	3,629 (46.0%)^a,c,d^	4,641 (52.1%)^a,b,d^	3,329 (64.7%)^a,b,c^	
Computation	1	2,413 (8.1%)	1,055 (13.8%)^b,c,d^	630 (8.0%)^a,c,d^	525 (5.9%)^a,b,d^	203 (3.9%)^a,b,c^	<0.001
	2	1907 (6.4%)	711 (9.3%)^b,c,d^	511 (6.5%)^a,c,d^	479 (5.4%)^a,b,d^	206 (4.0%)^a,b,c^	
	3	3,791 (12.8%)	1,226 (16.0%)^b,c,d^	1,040 (13.2%)^a,c,d^	1,061 (11.9%)^a,b,d^	464 (9.0%)^a,b,c^	
	4	7,351 (24.8%)	1936 (25.2%)^b,c,d^	2071 (26.2%)^a,c,d^	2,180 (24.5%)^a,b,d^	1,164 (22.6%)^a,b,c^	
	5	14,158 (47.8%)	2,742 (35.7%)^b,c,d^	3,645 (46.2%)^a,c,d^	4,659 (52.3%)^a,b,d^	3,112 (60.4%)^a,b,c^	
Drawing	0	11,267 (38.0%)	4,021 (52.4%)^b,c,d^	3,096 (39.2%)^a,c,d^	2,831 (31.8%)^a,b,d^	1,319 (25.6%)^a,b,c^	<0.001
	1	18,353 (62.0%)	3,649 (47.6%)^b,c,d^	4,801 (60.8%)^a,c,d^	6,073 (68.2%)^a,b,d^	3,830 (74.4%)^a,b,c^	

Superscript letters indicate significant differences between groups (*p* < 0.05), a: significant difference vs 1; b: significant difference vs 2; c: significant difference vs 3; d: significant difference vs 4. *p* < 0.05 denotes statistical significance. Group Illiterate: Illiterate; Group Primary: Primary school; Group Middle: Middle school; Group High: High school/vocational high school + Junior college or above.

Demographic analysis showed that the illiterate group was significantly older [62.0 (54.0–68.0) years] with a higher proportion of females (57.3%), while the high-education group was younger [58.0 (53.0–63.0) years] with more males (59.3%). Marital status was comparable across groups, with married individuals comprising over 87% in each group. The high-education group exhibited more favorable lifestyle characteristics, with significantly higher rates of Internet usage (38.0%) and social activity participation (65.4%) compared to other groups (*p* < 0.001).

Health status comparison revealed a higher chronic disease burden in the illiterate group, with significantly higher prevalence of hypertension (21.7%) and arthritis (29.4%). Cognitive function scores showed a clear educational gradient: the high-education group achieved the highest scores [20.0 (18.0–23.0)], while the illiterate group scored lowest [16.0 (13.0–18.0)]. This pattern was consistent across all cognitive domains. In calculation ability, 60.4% of the high-education group achieved maximum scores compared to 35.7% in the illiterate group. Pentagon-copying task completion rates were 74.4% and 47.6% for high-education and illiterate groups, respectively (Table 1).

### Analysis of influencing factors of cognitive function

**Figure 2:**
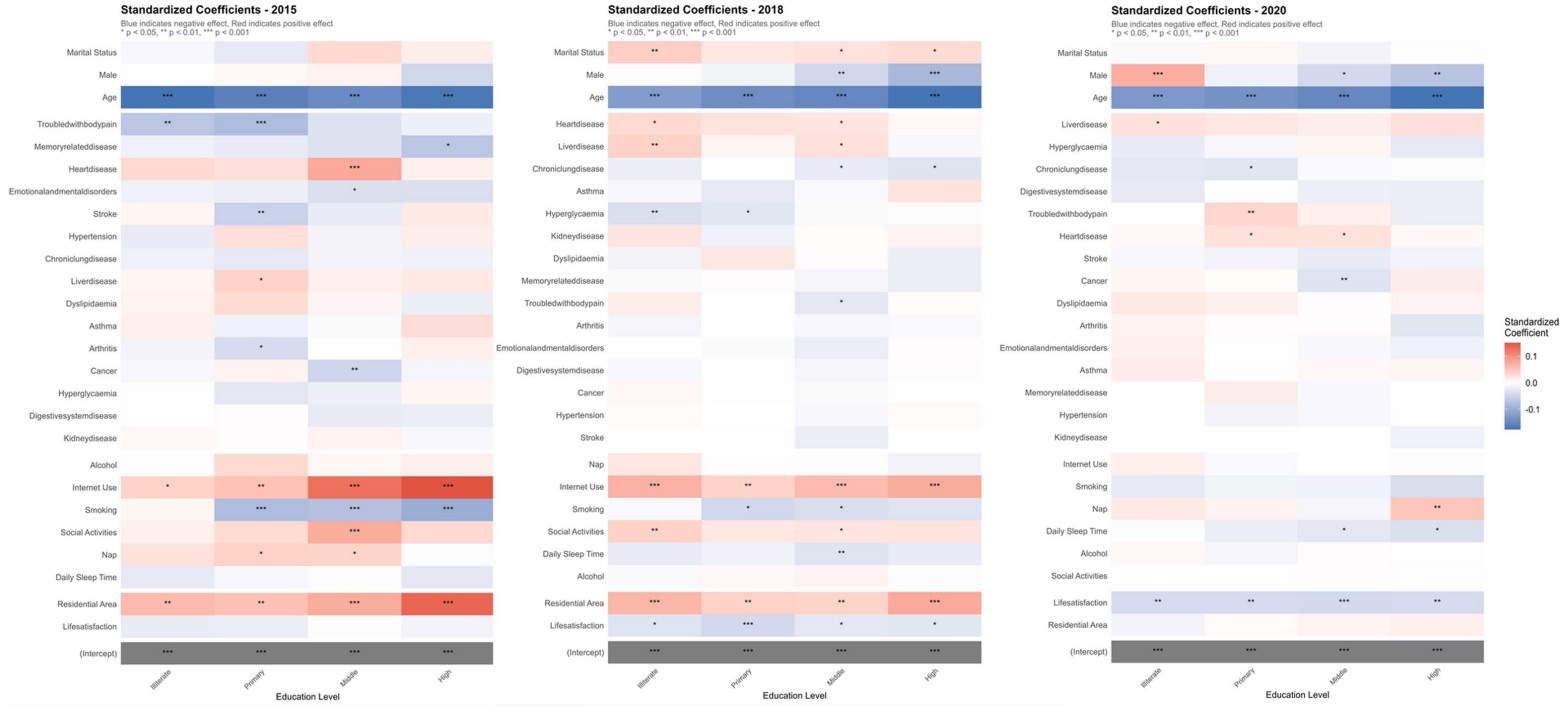
Correlation network of cognitive function and associated factors across educational groups. Red bubbles indicate positive correlations; green bubbles indicate negative correlations. Bubble size represents correlation strength. Group illiterate: illiterate; group primary: primary school; group middle: middle school; group high: high school/vocational high school + junior college or above. Significance levels: ^*^p < 0.05, **p < 0.01, ***p < 0.001.

Stratified Spearman correlation analysis identified education-moderated association patterns between cognitive function and candidate predictors across all four groups (Figure 2). Age exhibited the strongest and most consistent negative association, with correlation magnitude progressively intensifying across educational strata (illiterate: *r* = −0.156 to high-education: *r* = −0.276, all *p* < 0.001). Lifestyle factors including Internet use and smoking, as well as environmental factors such as urban residence, similarly demonstrated education-dependent association gradients. Health-related variables, including chronic diseases and physical pain, showed predominantly negative associations across all groups. Notably, gender and memory-related diseases displayed differential association patterns across educational strata. These crude association profiles, captured simultaneously across all predictor domains in Figure 2, provided preliminary evidence of educational effect modification and informed the multivariable linear regression and mixed-effects model analyses presented below.

**Figure 3:**
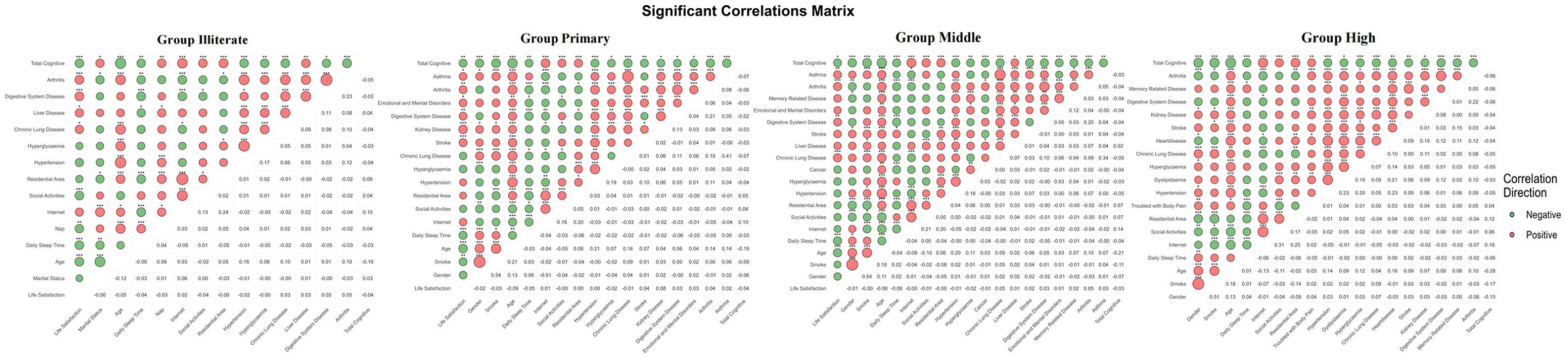
Standardized effects of various factors on cognitive function across education levels (2015–2020). Group illiterate: illiterate; Group primary: primary school; Group middle: middle school; Group high: high school/vocational high school + junior college or above. Significance levels: *p < 0.05, **p < 0.01, ***p < 0.001.

Multivariate analysis (Figure 3; Supplementary Table S1) further elucidated education-dependent patterns. Throughout 2015–2020, age consistently showed negative correlations with cognitive function (all *p* < 0.001), with the effect strengthening at higher educational levels. By 2020, the age effect coefficient in the high-education group (−0.109) substantially exceeded that of the illiterate group (−0.071).

Gender effects showed temporal and educational variation. In 2020, males demonstrated cognitive advantages in the illiterate group (*β* = 0.716, *p* < 0.001) but disadvantages in higher education groups (high-education group: *β* = −0.739, *p* < 0.001). Internet use maintained positive associations across all educational levels, particularly evident in 2018 and 2015 (*p* < 0.001). Smoking behavior showed negative impacts across all educational groups in 2015, most prominently in the high-education group (*β* = −0.689, *p* < 0.001).

Sleep duration demonstrated significant negative associations in medium-education and high-education groups by 2020 (medium-education: *β* = −0.345, *p* < 0.05; high-education: *β* = −0.500, *p* < 0.05). Cancer showed substantial negative impact in the medium-education group (2020: *β* = −1.964, *p* < 0.01), while memory-related diseases significantly affected the high-education group in 2015 (*β* = −2.325, *p* < 0.05). Life satisfaction demonstrated negative associations across all educational groups in 2020 (*p* < 0.01), while urban residence showed consistent positive effects across all educational groups in 2018 and 2015.

### Mixed-effects model analysis

The stepwise mixed-effects model analysis (Table 2) revealed distinctive patterns of key influencing factors across educational strata. Baseline cognitive function demonstrated a clear educational gradient in the intercept terms, progressively increasing from the illiterate group (15.888) to the high-education group (20.251, *p* < 0.001), indicating a robust positive association between educational attainment and baseline cognitive performance.

**Table 2:** Mixed effects models of cognitive function across educational groups.

	Group illiterate				Group primary				Group middle				Group high			
Variable	Model 1	Model 2	Model 3	Model 4	Model 1	Model 2	Model 3	Model 4	Model 1	Model 2	Model 3	Model 4	Model 1	Model 2	Model 3	Model 4
(Intercept)	15.888***	15.667***	15.677***	15.770***	17.545***	17.488***	17.520***	17.582***	18.612***	18.592***	18.547***	18.646***	20.251***	20.189***	20.035***	20.127***
Age	−0.661***	−0.668***	−0.676***	−0.665***	−0.697***	−0.664***	−0.683***	−0.666***	−0.922***	−0.869***	−0.889***	−0.878***	−1.106***	−1.024***	−1.052***	−1.033***
Male	0.148	0.127	0.118	0.125	−0.200*	−0.034	−0.032	−0.034	−0.357***	−0.173*	−0.175*	−0.167*	−0.676***	−0.449***	−0.453***	−0.450***
Married	0.049	0.006	0.016	0.012	0.110	0.078	0.071	0.083	0.114	0.071	0.079	0.080	0.153	0.079	0.064	0.054
Smoking		0.056	0.068	0.058		−0.418***	−0.414***	−0.394***		−0.460***	−0.439***	−0.432***		−0.668***	−0.614***	−0.607***
Alcohol		0.028	0.029	0.030		0.003	0.008	0.008		0.048	0.045	0.045		−0.015	−0.009	−0.006
Daily sleep time (≥ 8 h)		−0.105	−0.111	−0.112		−0.046	−0.054	−0.055		−0.152	−0.168*	−0.174*		−0.250*	−0.241*	−0.238*
Nap		0.255**	0.241**	0.234**		0.126	0.112	0.112		0.027	0.025	0.019		0.090	0.070	0.069
Internet use		0.299**	0.259*	0.254*		0.214*	0.176	0.169		0.324***	0.272**	0.267**		0.519***	0.416***	0.411***
Social activities		0.153*	0.144	0.140		0.150*	0.146	0.132		0.188**	0.188**	0.183*		0.185	0.177	0.179
Residential area (Urban)			0.192*	0.188*			0.174*	0.162			0.236**	0.221**			0.440***	0.439***
Life satisfaction (Dissatisfied)			−0.472***	−0.478***			−0.676***	−0.667***			−0.572***	−0.555***			−0.571**	−0.552**
Troubled with body pain				−0.030				0.002				−0.085				−0.026
Hypertension				−0.067				0.016				−0.142				0.008
Dyslipidaemia				0.144				0.415*				0.123				−0.093
Hyperglycaemia				−0.367				−0.511*				−0.014				−0.143
Cancer				0.173				0.236				−1.518**				0.357
Chronic lung disease				−0.318				−0.305				−0.286				−0.471
Liver disease				0.113*				0.083				0.113*				0.041
Heart disease				0.357*				0.380*				0.695***				0.173
Stroke				−0.086				−0.550				−0.940**				−0.435
Kidney disease				0.237				−0.259				0.079				0.010
Digestive system disease				−0.178				−0.005				−0.230				−0.166
Emotional and mental disorders				0.003				−0.604				−1.248*				−0.501
Memory related disease				−0.526				−0.000				−1.147*				−1.146
Arthritis				−0.108				−0.224*				−0.045				−0.139
Asthma				0.146				−0.243				−0.145				0.895

Values represent standardized coefficients (*p < 0.05, **p < 0.01, ***p < 0.001). Each model builds upon the previous one: The model presented in this table includes random intercepts for time and individual differences, Model 1: Demographics; Model 2: Model 1 + Lifestyle; Model 3: Model 2 + Environment; Model 4: Model 3 + Health Conditions. Group Illiterate: Illiterate; Group Primary: Primary school; Group Middle: Middle school; Group High: High school/vocational high school + Junior college or above.

Age-related cognitive decline was significant across all educational groups (*p* < 0.001), with the magnitude of decline intensifying at higher educational levels (illiterate: *β* = −0.665; high-education: *β* = −1.033). This pattern suggests an accelerated rate of cognitive decline among individuals with higher educational attainment.

Lifestyle factors showed education-dependent effects: smoking’s negative impact on cognition was more pronounced in higher education groups (high-education: *β* = −0.607, *p* < 0.001). Internet use demonstrated increasingly positive effects with higher educational levels (illiterate: *β* = 0.254; high-education: *β* = 0.411, *p* < 0.001). Notably, napping showed significant positive effects exclusively in the illiterate group (*β* = 0.234, *p* < 0.01).

Environmental factors also exhibited educational gradients. The positive impact of urban residence strengthened with increasing educational levels (illiterate: *β* = 0.188; high-education: *β* = 0.439, *p* < 0.001). Life satisfaction showed consistent negative associations across all educational strata (*β* range: −0.478 to −0.667, *p* < 0.001).

Health conditions demonstrated varying impacts across educational levels. Heart disease showed the strongest positive association in the medium-education group (*β* = 0.695, *p* < 0.001). Memory-related diseases exhibited significant negative impacts in the medium-education group (*β* = −1.147, *p* < 0.05), while cancer showed significant negative associations exclusively in this group (*β* = −1.518, *p* < 0.01).

These findings underscore the complex interplay between educational attainment and cognitive function determinants. While the high-education group exhibited accelerated age-related cognitive decline, they also demonstrated enhanced benefits from protective factors such as Internet use and urban residence.

### Sensitivity analysis

Alternative educational stratification schemes (Table 3; Supplementary Table S2) consistently confirmed the robust relationship between educational level and cognitive function. Under the three-level stratification scheme (Illiterate, Primary + Middle, High), baseline cognitive scores demonstrated a clear ascending pattern (15.770, 18.100, 20.127, *p* < 0.001). This gradient remained evident in the two-level stratification (Illiterate + Primary, Middle + High) (16.560, 19.124, *p* < 0.001).

**Table 3:** Mixed effects models of cognitive function across Two-Level educational stratification.

	Group Illiterate + Primary				Group Middle + High			
Variable	Model 1	Model 2	Model 3	Model 4	Model 1	Model 2	Model 3	Model 4
(Intercept)	16.646***	16.453***	16.464***	16.560***	19.182***	19.116***	19.011***	19.124***
Age	−0.742***	−0.733***	−0.747***	−0.732***	−0.958***	−0.891***	−0.918***	−0.904***
Male	0.101	0.151*	0.146*	0.149*	−0.439***	−0.233***	−0.236***	−0.234***
Married	0.066	0.024	0.029	0.030	0.132	0.066	0.073	0.069
Smoking		−0.120	−0.110	−0.105		−0.567***	−0.531***	−0.522***
Alcohol		0.031	0.035	0.037		0.032	0.032	0.032
Daily sleep time (≥ 8 h)		−0.076	−0.082	−0.084		−0.224**	−0.231***	−0.234***
Nap		0.216***	0.200***	0.197***		0.092	0.084	0.075
Internet use		0.271***	0.224**	0.219**		0.487***	0.395***	0.389***
Social activities		0.181***	0.173**	0.167**		0.206***	0.202***	0.199***
Residential area (Urban)			0.224***	0.214***			0.373***	0.359***
Life satisfaction (Dissatisfied)			−0.590***	−0.588***			−0.608***	−0.586***
Troubled with body pain				−0.025				−0.064
Hypertension				−0.052				−0.096
Dyslipidaemia				0.246*				0.142
Hyperglycaemia				−0.381*				−0.075
Cancer				0.334				−0.712
Chronic lung disease				−0.287*				−0.355*
Liver disease				0.098**				0.094**
Heart disease				0.443***				0.526***
Stroke				−0.305				−0.720**
Kidney disease				−0.014				0.093
Digestive system disease				−0.103				−0.249*
Emotional and mental disorders				−0.345				−1.046*
Memory related disease				−0.329				−1.073**
Arthritis				−0.228**				−0.177
Asthma				−0.000				0.091

Values represent standardized coefficients (*p < 0.05, **p < 0.01, ***p < 0.001). Each model builds upon the previous one: The model presented in this table includes random intercepts for time and individual differences, Model 1: Demographics; Model 2: Model 1 + Lifestyle; Model 3: Model 2 + Environment; Model 4: Model 3 + Health Conditions. Group Illiterate: Illiterate; Group Primary: Primary school; Group Middle: Middle school; Group High: High school/vocational high school + Junior college or above.

Age-related effects further corroborated our primary findings: cognitive decline intensified with increasing educational level. The three-level stratification revealed progressively stronger age effects from illiterate to high-education groups (−0.665, −0.824, −1.033, *p* < 0.001), with this pattern remaining significant in the two-level classification.

Among lifestyle factors, Internet use maintained its education-dependent protective effect, showing strongest benefits in the high-education group (*β* = 0.411, *p* < 0.001). Similarly, smoking’s negative impact demonstrated increased magnitude at higher educational levels. Social and environmental factors maintained consistent patterns across stratification schemes, with urban residence and life satisfaction effects remaining stable across alternative groupings.

Health status effects demonstrated robust patterns: heart disease showed maximal positive association in the Primary + Middle group (*β* = 0.545, *p* < 0.001). Mental and emotional disorders and stroke exhibited their strongest negative impacts within this same group (*β* = −0.941, *p* < 0.05; *β* = −0.742, *p* < 0.01, respectively).

These sensitivity analyses not only validated our primary findings but also reinforced the significance of educational level as a key moderator of cognitive function determinants. The consistency across different stratification approaches strengthens the reliability of our main conclusions regarding education’s role in cognitive function modulation.

Formal interaction testing in the pooled model provided additional statistical support for the stratified findings. Educational level showed statistically significant overall modification of the associations of age [*F*(3) = 9.86, *p* < 0.001], sex [F(3) = 16.25, *p* < 0.001], and internet use [F(3) = 3.40, *p* = 0.017] with cognitive function, while urban residence and memory-related disease did not reach significance at this level. The direction of all significant interaction contrasts was broadly consistent with the stratified estimates in Table 2, suggesting that the observed between-group differences are likely to reflect genuine effect modification rather than chance findings (Supplementary Table S3).

## Discussion

This large-scale longitudinal study, drawing on nationally representative CHARLS data spanning 2015–2020, systematically examined the moderating role of educational attainment in cognitive function determinants among middle-aged and older Chinese adults. Our findings suggest that educational level not only independently associates with cognitive performance but may also modulate its relationships with gender differences, Internet use, residential environment, and chronic disease burden.

Among the observed education-dependent patterns, the reversal of the gender-cognition association across educational strata is particularly striking. In the illiterate group, male sex corresponded with relatively higher cognitive scores, yet this direction appeared to invert among those with middle or higher education. This complexity is not entirely unexpected. Female superiority in verbal memory has been repeatedly documented, persisting even at advanced ages (Arenaza Urquijo et al., 2024; Marseglia et al., 2024), and some evidence points to a stronger association between college-level education and executive function preservation specifically among women (Lam et al., 2024). Within lower-education cohorts, the cognitive demands inherent in domestic responsibilities and community-level social roles may have afforded women a source of ongoing mental engagement—an informal pathway to cognitive maintenance that resonates with documented patterns of female neuropathological resilience (Guo et al., 2023). Conversely, in higher-education groups, longstanding disparities in occupational access and schooling quality between men and women may have resulted in unequal cognitive reserve accumulation despite nominally similar attainment levels (Arenaza Urquijo et al., 2024). These interpretations, however, rest on unmeasured constructs and cross-sectional comparisons within the data, and should be regarded as tentative pending more targeted investigation.

Across all educational subgroups, Internet use carried a positive association with cognitive function, with the magnitude of this association appearing to increase alongside educational attainment. Prior evidence has linked digital engagement to a lower likelihood of mild cognitive impairment (Li et al., 2022), and educational background has emerged as a meaningful correlate of how older adults engage with online environments (Macdonald and Hülür, 2021). The relationship between digital exclusion and cognitive disadvantage also appears socioeconomically patterned (Wang et al., 2024), and individuals with higher educational attainment tend to show both more active internet engagement and more favorable cognitive profiles (Afsar, 2013). A plausible reading of the education-graded association is that it captures variation in the cognitive intensity of online activity rather than differences in connectivity per se—higher-educated users may be more inclined toward information-seeking and learning-oriented tasks. That said, the binary measurement of internet use in this study precludes any examination of frequency or content, and reverse causation—whereby better cognitive function facilitates internet adoption—cannot be ruled out from observational data alone. The pattern nonetheless raises the possibility that digital inclusion initiatives for educationally disadvantaged older adults may need to prioritize skill development alongside access.

A particularly counterintuitive observation concerns memory-related disease, whose negative association with cognitive function was most pronounced in the high-education group. Rather than contradicting cognitive reserve theory, this finding sits comfortably within threshold-based formulations (Ophey et al., 2024; Guzzetti et al., 2019), which hold that compensatory neural processes may sustain function in the face of accumulating pathology—but only up to a point, beyond which decline may proceed more rapidly in those with higher reserve. Whether the pattern here reflects this compensatory ceiling effect or instead mirrors systematic differences in disease presentation or severity across educational groups remains an open question. At minimum, the observation cautions against treating high educational attainment as a reliable indicator of cognitive resilience in the presence of memory-related conditions, and points to the value of vigilant screening in this population.

Urban residence was associated with higher cognitive scores across all educational groups, though the strength of this association appeared to scale with educational level—a finding broadly in line with research linking urban living to more cognitively stimulating environments for older adults (Miura et al., 2023). The capacity to translate the structural advantages of urban settings into health gains has been linked to individual human capital (Yuan et al., 2023), while community-level social infrastructure may partially buffer against the cognitive costs of urban stressors (Tian et al., 2024). Whether the education-dependent amplification of the urban association observed here reflects active resource utilization, residential selection, or residual confounding is difficult to assess within the current observational framework.

The main strengths of this study are as follows: Firstly, based on large-sample and multi-time-point data, it can better control confounding factors and observe time trends. Secondly, the mixed-effects model was used to consider individual differences and time effects. Thirdly, the robustness of the results was verified through sensitivity analysis of multiple grouping schemes.

This study has several methodological considerations that limit the interpretation of these findings. Participant continuity across all three waves was incomplete, introducing the possibility of selective attrition and cohort-related bias. The cognitive instrument, though widely adopted in aging research, covers a restricted set of domains and may lack the sensitivity to detect early or domain-specific changes. The binary operationalization of internet use represents a notable constraint, collapsing variation in frequency, purpose, and proficiency into a single indicator. Beyond these measurement issues, confounding by variables not captured in CHARLS—among them physical activity patterns, nutritional intake, and finer-grained socioeconomic indicators—cannot be adequately controlled. These considerations suggest that the present findings are more appropriately viewed as generating hypotheses than as establishing firm conclusions, and that causal inference would require evidence from experimental or natural experiment designs.

Based on the research findings, we suggest: (1) Formulating differential cognitive function maintenance strategies for populations with different educational levels, with particular attention to the education-dependency of gender differences. (2) Strengthening digital skills training for people with a low educational level and attaching importance to the protective effect of Internet use on cognitive function. (3) Considering differences in educational level in chronic disease management and carrying out targeted health education. (4) Designing social participation forms suitable for different educational levels to improve participation.

Future research suggestions: (1) Conducting longer-term follow-up studies to deeply explore the causal relationships of influencing factors. (2) Using more refined cognitive function assessment tools. (3) Exploring the interaction between educational level and other socioeconomic factors. (4) Conducting intervention studies to verify the practical value of the findings of this study.

## Conclusion

Drawing on longitudinal CHARLS data, this study suggests that educational attainment may serve as a meaningful modifier of the factors associated with cognitive function in older Chinese adults. Age-related cognitive decline appeared more pronounced at higher educational levels, the gender-cognition association shifted across strata, and the benefits of Internet use and urban residence both appeared to strengthen alongside educational attainment, while memory-related disease carried a particularly notable negative association in the high-education group. These observations point to the potential value of incorporating educational background into the design of targeted cognitive health initiatives.

## Data Availability

The raw data supporting the conclusions of this article will be made available by the authors, without undue reservation.
