# Supplementary material for: Analysis of disparate factors affecting cognitive function among populations with different educational levels: a large-scale longitudinal study
Source: Front Psychol. 2026 Mar 19;17:1564721. doi: 10.3389/fpsyg.2026.1564721 (PMC13043331; doi:10.3389/fpsyg.2026.1564721)
Supplement: Supplementary file 1 [file table_1.docx]

eTable 1. Effects of Various Factors on Cognitive Function Across Education Levels (2015-2020).

| **Variable (2020)** | **Estimate** | **Standardization coefficient** | **Lower 95%CI** | **Upper 95%CI** | **Education Group** | ***P*** |
| --- | --- | --- | --- | --- | --- | --- |
| Age | -0.071 | -0.180 | -0.086 | -0.056 | Illiterate | <0.001*** |
| Age | -0.069 | -0.191 | -0.083 | -0.055 | Primary | <0.001*** |
| Age | -0.094 | -0.212 | -0.110 | -0.078 | Middle | <0.001*** |
| Age | -0.109 | -0.249 | -0.129 | -0.089 | High | <0.001*** |
| Male | 0.716 | 0.103 | 0.329 | 1.103 | Illiterate | <0.001*** |
| Male | -0.158 | -0.023 | -0.539 | 0.224 | Primary | 0.417 |
| Male | -0.468 | -0.066 | -0.836 | -0.099 | Middle | 0.013* |
| Male | -0.739 | -0.102 | -1.183 | -0.296 | High | 0.001** |
| Marital Status | -0.072 | -0.008 | -0.420 | 0.276 | Illiterate | 0.685 |
| Marital Status | 0.093 | 0.010 | -0.254 | 0.439 | Primary | 0.601 |
| Marital Status | -0.175 | -0.019 | -0.499 | 0.148 | Middle | 0.288 |
| Marital Status | 0.036 | 0.004 | -0.379 | 0.452 | High | 0.865 |
| Arthritis | 0.118 | 0.015 | -0.182 | 0.418 | Illiterate | 0.440 |
| Arthritis | 0.052 | 0.006 | -0.266 | 0.370 | Primary | 0.749 |
| Arthritis | 0.055 | 0.006 | -0.263 | 0.373 | Middle | 0.735 |
| Arthritis | -0.425 | -0.044 | -0.872 | 0.022 | High | 0.062 |
| Asthma | 0.483 | 0.025 | -0.326 | 1.292 | Illiterate | 0.242 |
| Asthma | 0.085 | 0.004 | -0.688 | 0.859 | Primary | 0.829 |
| Asthma | 0.209 | 0.008 | -0.689 | 1.107 | Middle | 0.648 |
| Asthma | 0.356 | 0.013 | -0.934 | 1.646 | High | 0.589 |
| Cancer | 0.407 | 0.012 | -0.882 | 1.696 | Illiterate | 0.536 |
| Cancer | 0.227 | 0.007 | -0.930 | 1.385 | Primary | 0.700 |
| Cancer | -1.964 | -0.047 | -3.393 | -0.536 | Middle | 0.007** |
| Cancer | 0.949 | 0.025 | -0.736 | 2.634 | High | 0.269 |
| Chronic lung disease | -0.493 | -0.040 | -1.006 | 0.020 | Illiterate | 0.060 |
| Chronic lung disease | -0.564 | -0.044 | -1.083 | -0.045 | Primary | 0.033* |
| Chronic lung disease | -0.168 | -0.011 | -0.737 | 0.401 | Middle | 0.563 |
| Chronic lung disease | -0.104 | -0.007 | -0.833 | 0.625 | High | 0.780 |
| Digestive system disease | -0.326 | -0.037 | -0.663 | 0.011 | Illiterate | 0.058 |
| Digestive system disease | -0.017 | -0.002 | -0.361 | 0.326 | Primary | 0.922 |
| Digestive system disease | -0.245 | -0.025 | -0.589 | 0.100 | Middle | 0.164 |
| Digestive system disease | -0.313 | -0.031 | -0.779 | 0.153 | High | 0.188 |
| Dyslipidaemia | 0.340 | 0.028 | -0.134 | 0.814 | Illiterate | 0.159 |
| Dyslipidaemia | 0.260 | 0.021 | -0.228 | 0.748 | Primary | 0.297 |
| Dyslipidaemia | 0.062 | 0.005 | -0.353 | 0.476 | Middle | 0.770 |
| Dyslipidaemia | 0.153 | 0.015 | -0.310 | 0.615 | High | 0.518 |
| Emotional and mental disorders | 0.882 | 0.022 | -0.632 | 2.397 | Illiterate | 0.253 |
| Emotional and mental disorders | 0.030 | 0.001 | -1.636 | 1.695 | Primary | 0.972 |
| Emotional and mental disorders | -0.537 | -0.011 | -2.173 | 1.099 | Middle | 0.520 |
| Emotional and mental disorders | -1.047 | -0.022 | -3.139 | 1.045 | High | 0.326 |
| Heart disease | 0.119 | 0.010 | -0.343 | 0.580 | Illiterate | 0.614 |
| Heart disease | 0.471 | 0.039 | 0.001 | 0.941 | Primary | 0.049* |
| Heart disease | 0.474 | 0.038 | 0.023 | 0.925 | Middle | 0.039* |
| Heart disease | 0.104 | 0.009 | -0.447 | 0.654 | High | 0.712 |
| Hyperglycaemia | -0.525 | -0.034 | -1.116 | 0.065 | Illiterate | 0.081 |
| Hyperglycaemia | -0.174 | -0.011 | -0.762 | 0.414 | Primary | 0.562 |
| Hyperglycaemia | 0.208 | 0.013 | -0.364 | 0.779 | Middle | 0.476 |
| Hyperglycaemia | -0.515 | -0.035 | -1.174 | 0.143 | High | 0.125 |
| Hypertension | 0.008 | 0.001 | -0.327 | 0.343 | Illiterate | 0.963 |
| Hypertension | -0.184 | -0.020 | -0.541 | 0.173 | Primary | 0.313 |
| Hypertension | -0.111 | -0.012 | -0.454 | 0.232 | Middle | 0.525 |
| Hypertension | -0.014 | -0.002 | -0.438 | 0.409 | High | 0.948 |
| Kidney disease | 0.049 | 0.003 | -0.536 | 0.635 | Illiterate | 0.868 |
| Kidney disease | -0.017 | -0.001 | -0.628 | 0.595 | Primary | 0.957 |
| Kidney disease | 0.002 | 0.000 | -0.619 | 0.624 | Middle | 0.994 |
| Kidney disease | -0.396 | -0.024 | -1.123 | 0.331 | High | 0.285 |
| Liver disease | 0.784 | 0.040 | 0.045 | 1.522 | Illiterate | 0.037* |
| Liver disease | 0.632 | 0.030 | -0.161 | 1.426 | Primary | 0.118 |
| Liver disease | 0.475 | 0.024 | -0.215 | 1.166 | Middle | 0.177 |
| Liver disease | 0.811 | 0.042 | -0.052 | 1.674 | High | 0.065 |
| Memory related disease | -0.070 | -0.002 | -1.394 | 1.254 | Illiterate | 0.917 |
| Memory related disease | 1.015 | 0.023 | -0.617 | 2.646 | Primary | 0.223 |
| Memory related disease | -0.503 | -0.011 | -2.053 | 1.046 | Middle | 0.524 |
| Memory related disease | 0.002 | 0.000 | -1.845 | 1.848 | High | 0.999 |
| Stroke | -0.321 | -0.014 | -1.176 | 0.534 | Illiterate | 0.461 |
| Stroke | -0.429 | -0.018 | -1.318 | 0.460 | Primary | 0.344 |
| Stroke | -0.872 | -0.031 | -1.819 | 0.075 | Middle | 0.071 |
| Stroke | -0.534 | -0.021 | -1.667 | 0.600 | High | 0.356 |
| Troubled with body pain | 0.014 | 0.002 | -0.256 | 0.285 | Illiterate | 0.917 |
| Troubled with body pain | 0.373 | 0.054 | 0.105 | 0.640 | Primary | 0.006** |
| Troubled with body pain | 0.141 | 0.020 | -0.108 | 0.391 | Middle | 0.268 |
| Troubled with body pain | -0.205 | -0.029 | -0.522 | 0.112 | High | 0.206 |
| Alcohol | 0.065 | 0.010 | -0.186 | 0.317 | Illiterate | 0.609 |
| Alcohol | -0.107 | -0.015 | -0.361 | 0.148 | Primary | 0.410 |
| Alcohol | 0.042 | 0.006 | -0.200 | 0.285 | Middle | 0.732 |
| Alcohol | -0.024 | -0.003 | -0.336 | 0.288 | High | 0.879 |
| Daily Sleep Time | -0.035 | -0.004 | -0.334 | 0.264 | Illiterate | 0.818 |
| Daily Sleep Time | -0.233 | -0.029 | -0.534 | 0.067 | Primary | 0.128 |
| Daily Sleep Time | -0.345 | -0.040 | -0.637 | -0.052 | Middle | 0.021* |
| Daily Sleep Time | -0.500 | -0.055 | -0.897 | -0.102 | High | 0.014* |
| Internet Use | 0.144 | 0.021 | -0.127 | 0.414 | Illiterate | 0.298 |
| Internet Use | -0.066 | -0.009 | -0.339 | 0.207 | Primary | 0.637 |
| Internet Use | -0.010 | -0.001 | -0.269 | 0.250 | Middle | 0.941 |
| Internet Use | -0.035 | -0.005 | -0.372 | 0.302 | High | 0.837 |
| Nap | 0.188 | 0.027 | -0.067 | 0.443 | Illiterate | 0.149 |
| Nap | 0.125 | 0.018 | -0.138 | 0.388 | Primary | 0.351 |
| Nap | -0.108 | -0.015 | -0.359 | 0.143 | Middle | 0.398 |
| Nap | 0.558 | 0.073 | 0.221 | 0.895 | High | 0.001** |
| Smoking | -0.247 | -0.036 | -0.631 | 0.137 | Illiterate | 0.207 |
| Smoking | -0.131 | -0.019 | -0.507 | 0.245 | Primary | 0.494 |
| Smoking | -0.166 | -0.024 | -0.524 | 0.193 | Middle | 0.365 |
| Smoking | -0.408 | -0.058 | -0.830 | 0.014 | High | 0.058 |
| Social Activities | 0.023 | 0.003 | -0.231 | 0.277 | Illiterate | 0.857 |
| Social Activities | 0.020 | 0.003 | -0.236 | 0.277 | Primary | 0.877 |
| Social Activities | 0.039 | 0.006 | -0.204 | 0.282 | Middle | 0.753 |
| Social Activities | 0.027 | 0.004 | -0.287 | 0.340 | High | 0.868 |
| Residential Area | -0.127 | -0.018 | -0.396 | 0.142 | Illiterate | 0.354 |
| Residential Area | 0.051 | 0.007 | -0.219 | 0.321 | Primary | 0.712 |
| Residential Area | 0.104 | 0.015 | -0.147 | 0.356 | Middle | 0.416 |
| Residential Area | 0.159 | 0.022 | -0.174 | 0.492 | High | 0.348 |
| Life Satisfaction | -0.702 | -0.062 | -1.125 | -0.279 | Illiterate | 0.001** |
| Life Satisfaction | -0.723 | -0.060 | -1.172 | -0.274 | Primary | 0.002** |
| Life Satisfaction | -0.847 | -0.064 | -1.298 | -0.396 | Middle | <0.001*** |
| Life Satisfaction | -0.853 | -0.059 | -1.495 | -0.212 | High | 0.009** |
| (Intercept) | 22.837 | 22.837 | 19.874 | 25.800 | Illiterate | <0.001*** |
| (Intercept) | 24.178 | 24.178 | 20.957 | 27.399 | Primary | <0.001*** |
| (Intercept) | 22.584 | 22.584 | 19.275 | 25.894 | Middle | <0.001*** |
| (Intercept) | 26.677 | 26.677 | 22.677 | 30.676 | High | <0.001*** |
| **Variable (2018)** | **Estimate** | **Standardization coefficient** | **Lower 95%CI** | **Upper 95%CI** | **Education Group** | ***P*** |
| Age | -0.077 | -0.184 | -0.095 | -0.058 | Illiterate | <0.001*** |
| Age | -0.086 | -0.216 | -0.103 | -0.070 | Primary | <0.001*** |
| Age | -0.110 | -0.226 | -0.128 | -0.092 | Middle | <0.001*** |
| Age | -0.125 | -0.265 | -0.145 | -0.104 | High | <0.001*** |
| Male | 0.039 | 0.005 | -0.431 | 0.509 | Illiterate | 0.871 |
| Male | -0.158 | -0.021 | -0.580 | 0.265 | Primary | 0.464 |
| Male | -0.546 | -0.070 | -0.933 | -0.158 | Middle | 0.006** |
| Male | -1.048 | -0.130 | -1.504 | -0.593 | High | <0.001*** |
| Marital Status | 0.777 | 0.066 | 0.284 | 1.270 | Illiterate | 0.002** |
| Marital Status | 0.443 | 0.034 | -0.051 | 0.937 | Primary | 0.078 |
| Marital Status | 0.592 | 0.041 | 0.107 | 1.078 | Middle | 0.017* |
| Marital Status | 0.709 | 0.050 | 0.128 | 1.290 | High | 0.017* |
| Arthritis | -0.141 | -0.017 | -0.505 | 0.223 | Illiterate | 0.448 |
| Arthritis | -0.022 | -0.002 | -0.372 | 0.329 | Primary | 0.904 |
| Arthritis | -0.113 | -0.012 | -0.446 | 0.220 | Middle | 0.505 |
| Arthritis | -0.109 | -0.010 | -0.565 | 0.347 | High | 0.639 |
| Asthma | -0.252 | -0.013 | -1.184 | 0.680 | Illiterate | 0.596 |
| Asthma | -0.777 | -0.038 | -1.584 | 0.031 | Primary | 0.059 |
| Asthma | -0.210 | -0.008 | -1.085 | 0.664 | Middle | 0.637 |
| Asthma | 1.117 | 0.038 | -0.176 | 2.411 | High | 0.090 |
| Cancer | 0.460 | 0.012 | -1.169 | 2.090 | Illiterate | 0.580 |
| Cancer | 0.090 | 0.003 | -1.212 | 1.393 | Primary | 0.892 |
| Cancer | -0.486 | -0.010 | -2.111 | 1.140 | Middle | 0.558 |
| Cancer | 0.080 | 0.002 | -1.656 | 1.817 | High | 0.928 |
| Chronic lung disease | -0.421 | -0.031 | -1.055 | 0.213 | Illiterate | 0.193 |
| Chronic lung disease | -0.022 | -0.002 | -0.578 | 0.534 | Primary | 0.937 |
| Chronic lung disease | -0.568 | -0.036 | -1.124 | -0.012 | Middle | 0.045* |
| Chronic lung disease | -0.798 | -0.049 | -1.513 | -0.082 | High | 0.029* |
| Digestive system disease | -0.114 | -0.012 | -0.519 | 0.291 | Illiterate | 0.581 |
| Digestive system disease | 0.011 | 0.001 | -0.366 | 0.387 | Primary | 0.955 |
| Digestive system disease | -0.194 | -0.018 | -0.555 | 0.168 | Middle | 0.293 |
| Digestive system disease | -0.087 | -0.008 | -0.555 | 0.382 | High | 0.716 |
| Dyslipidaemia | -0.250 | -0.021 | -0.788 | 0.289 | Illiterate | 0.364 |
| Dyslipidaemia | 0.438 | 0.033 | -0.096 | 0.973 | Primary | 0.108 |
| Dyslipidaemia | 0.080 | 0.006 | -0.357 | 0.516 | Middle | 0.721 |
| Dyslipidaemia | -0.335 | -0.031 | -0.793 | 0.123 | High | 0.152 |
| Emotional and mental disorders | 0.115 | 0.003 | -1.403 | 1.632 | Illiterate | 0.882 |
| Emotional and mental disorders | -0.422 | -0.010 | -2.040 | 1.196 | Primary | 0.609 |
| Emotional and mental disorders | -1.392 | -0.031 | -2.890 | 0.106 | Middle | 0.069 |
| Emotional and mental disorders | 0.394 | 0.007 | -1.818 | 2.607 | High | 0.727 |
| Heart disease | 0.660 | 0.054 | 0.120 | 1.199 | Illiterate | 0.017* |
| Heart disease | 0.468 | 0.037 | -0.022 | 0.957 | Primary | 0.061 |
| Heart disease | 0.468 | 0.035 | 0.004 | 0.932 | Middle | 0.048* |
| Heart disease | 0.128 | 0.010 | -0.399 | 0.656 | High | 0.633 |
| Hyperglycaemia | -0.963 | -0.058 | -1.673 | -0.252 | Illiterate | 0.008** |
| Hyperglycaemia | -0.787 | -0.047 | -1.433 | -0.142 | Primary | 0.017* |
| Hyperglycaemia | -0.155 | -0.009 | -0.733 | 0.424 | Middle | 0.600 |
| Hyperglycaemia | -0.073 | -0.005 | -0.710 | 0.565 | High | 0.823 |
| Hypertension | 0.072 | 0.008 | -0.325 | 0.470 | Illiterate | 0.721 |
| Hypertension | 0.004 | 0.000 | -0.384 | 0.392 | Primary | 0.984 |
| Hypertension | -0.124 | -0.012 | -0.476 | 0.228 | Middle | 0.489 |
| Hypertension | 0.083 | 0.008 | -0.349 | 0.515 | High | 0.706 |
| Kidney disease | 0.573 | 0.035 | -0.108 | 1.254 | Illiterate | 0.099 |
| Kidney disease | -0.405 | -0.023 | -1.066 | 0.256 | Primary | 0.230 |
| Kidney disease | 0.156 | 0.008 | -0.472 | 0.784 | Middle | 0.625 |
| Kidney disease | 0.281 | 0.016 | -0.430 | 0.992 | High | 0.439 |
| Liver disease | 1.272 | 0.062 | 0.413 | 2.132 | Illiterate | 0.004** |
| Liver disease | 0.277 | 0.013 | -0.531 | 1.085 | Primary | 0.501 |
| Liver disease | 0.902 | 0.041 | 0.179 | 1.626 | Middle | 0.014* |
| Liver disease | -0.297 | -0.015 | -1.110 | 0.516 | High | 0.474 |
| Memory related disease | -0.373 | -0.009 | -2.032 | 1.286 | Illiterate | 0.659 |
| Memory related disease | 0.314 | 0.007 | -1.361 | 1.989 | Primary | 0.713 |
| Memory related disease | -0.858 | -0.017 | -2.510 | 0.793 | Middle | 0.308 |
| Memory related disease | -1.178 | -0.032 | -2.706 | 0.351 | High | 0.131 |
| Stroke | -0.044 | -0.002 | -1.036 | 0.949 | Illiterate | 0.931 |
| Stroke | -0.026 | -0.001 | -1.072 | 1.021 | Primary | 0.962 |
| Stroke | -0.853 | -0.031 | -1.754 | 0.048 | Middle | 0.063 |
| Stroke | -0.061 | -0.002 | -1.131 | 1.009 | High | 0.911 |
| Troubled with body pain | 0.185 | 0.024 | -0.137 | 0.507 | Illiterate | 0.259 |
| Troubled with body pain | -0.002 | 0.000 | -0.300 | 0.295 | Primary | 0.988 |
| Troubled with body pain | -0.331 | -0.043 | -0.595 | -0.067 | Middle | 0.014* |
| Troubled with body pain | 0.065 | 0.008 | -0.258 | 0.389 | High | 0.692 |
| Alcohol | -0.069 | -0.009 | -0.372 | 0.234 | Illiterate | 0.656 |
| Alcohol | 0.089 | 0.011 | -0.195 | 0.372 | Primary | 0.540 |
| Alcohol | 0.120 | 0.016 | -0.135 | 0.375 | Middle | 0.355 |
| Alcohol | -0.047 | -0.006 | -0.362 | 0.268 | High | 0.769 |
| Daily Sleep Time | -0.294 | -0.035 | -0.634 | 0.047 | Illiterate | 0.091 |
| Daily Sleep Time | -0.197 | -0.023 | -0.513 | 0.118 | Primary | 0.220 |
| Daily Sleep Time | -0.423 | -0.049 | -0.712 | -0.134 | Middle | 0.004** |
| Daily Sleep Time | -0.340 | -0.035 | -0.727 | 0.047 | High | 0.085 |
| Internet Use | 1.489 | 0.109 | 0.898 | 2.081 | Illiterate | <0.001*** |
| Internet Use | 0.706 | 0.060 | 0.242 | 1.170 | Primary | 0.003** |
| Internet Use | 0.827 | 0.092 | 0.494 | 1.159 | Middle | <0.001*** |
| Internet Use | 0.910 | 0.116 | 0.536 | 1.284 | High | <0.001*** |
| Nap | 0.231 | 0.032 | -0.070 | 0.533 | Illiterate | 0.132 |
| Nap | 0.031 | 0.004 | -0.251 | 0.313 | Primary | 0.829 |
| Nap | 0.044 | 0.006 | -0.212 | 0.300 | Middle | 0.736 |
| Nap | -0.154 | -0.019 | -0.480 | 0.172 | High | 0.355 |
| Smoking | -0.086 | -0.012 | -0.550 | 0.377 | Illiterate | 0.716 |
| Smoking | -0.546 | -0.072 | -0.959 | -0.133 | Primary | 0.01* |
| Smoking | -0.477 | -0.062 | -0.850 | -0.104 | Middle | 0.012* |
| Smoking | -0.390 | -0.050 | -0.812 | 0.032 | High | 0.070 |
| Social Activities | 0.467 | 0.064 | 0.155 | 0.779 | Illiterate | 0.003** |
| Social Activities | 0.244 | 0.032 | -0.053 | 0.541 | Primary | 0.107 |
| Social Activities | 0.291 | 0.036 | 0.002 | 0.581 | Middle | 0.049* |
| Social Activities | 0.341 | 0.038 | -0.078 | 0.760 | High | 0.110 |
| Residential Area | 0.925 | 0.102 | 0.552 | 1.299 | Illiterate | <0.001*** |
| Residential Area | 0.566 | 0.065 | 0.240 | 0.893 | Primary | 0.001** |
| Residential Area | 0.466 | 0.059 | 0.197 | 0.735 | Middle | 0.001** |
| Residential Area | 0.963 | 0.119 | 0.623 | 1.303 | High | <0.001*** |
| Life Satisfaction | -0.603 | -0.050 | -1.102 | -0.104 | Illiterate | 0.018* |
| Life Satisfaction | -0.879 | -0.068 | -1.357 | -0.402 | Primary | <0.001*** |
| Life Satisfaction | -0.560 | -0.041 | -1.013 | -0.108 | Middle | 0.015* |
| Life Satisfaction | -0.710 | -0.046 | -1.332 | -0.088 | High | 0.025* |
| (Intercept) | 23.863 | 23.863 | 20.472 | 27.254 | Illiterate | <0.001*** |
| (Intercept) | 24.058 | 24.058 | 20.750 | 27.367 | Primary | <0.001*** |
| (Intercept) | 23.006 | 23.006 | 19.678 | 26.334 | Middle | <0.001*** |
| (Intercept) | 28.787 | 28.787 | 24.912 | 32.661 | High | <0.001*** |
| **Variable (2015)** | **Estimate** | **Standardization coefficient** | **Lower 95%CI** | **Upper 95%CI** | **Education Group** | ***P*** |
| Age | -0.067 | -0.176 | -0.082 | -0.052 | Illiterate | <0.001*** |
| Age | -0.057 | -0.159 | -0.072 | -0.041 | Primary | <0.001*** |
| Age | -0.061 | -0.146 | -0.078 | -0.044 | Middle | <0.001*** |
| Age | -0.073 | -0.171 | -0.097 | -0.049 | High | <0.001*** |
| Male | 0.011 | 0.002 | -0.229 | 0.251 | Illiterate | 0.928 |
| Male | 0.049 | 0.007 | -0.209 | 0.306 | Primary | 0.712 |
| Male | 0.084 | 0.012 | -0.167 | 0.335 | Middle | 0.512 |
| Male | -0.316 | -0.047 | -0.662 | 0.030 | High | 0.073 |
| Marital Status | -0.103 | -0.010 | -0.491 | 0.285 | Illiterate | 0.602 |
| Marital Status | -0.267 | -0.025 | -0.682 | 0.148 | Primary | 0.207 |
| Marital Status | 0.378 | 0.034 | -0.037 | 0.792 | Middle | 0.074 |
| Marital Status | 0.187 | 0.016 | -0.410 | 0.783 | High | 0.540 |
| Arthritis | -0.089 | -0.013 | -0.356 | 0.177 | Illiterate | 0.512 |
| Arthritis | -0.313 | -0.043 | -0.609 | -0.018 | Primary | 0.038* |
| Arthritis | 0.015 | 0.002 | -0.289 | 0.319 | Middle | 0.923 |
| Arthritis | 0.128 | 0.015 | -0.332 | 0.589 | High | 0.584 |
| Asthma | 0.269 | 0.016 | -0.422 | 0.960 | Illiterate | 0.445 |
| Asthma | -0.252 | -0.016 | -0.924 | 0.421 | Primary | 0.463 |
| Asthma | -0.125 | -0.006 | -0.966 | 0.717 | Middle | 0.772 |
| Asthma | 0.674 | 0.031 | -0.594 | 1.942 | High | 0.297 |
| Cancer | -0.364 | -0.010 | -1.770 | 1.043 | Illiterate | 0.612 |
| Cancer | 0.358 | 0.011 | -0.918 | 1.633 | Primary | 0.582 |
| Cancer | -2.321 | -0.051 | -4.022 | -0.621 | Middle | 0.007** |
| Cancer | -0.487 | -0.012 | -2.473 | 1.500 | High | 0.631 |
| Chronic lung disease | -0.178 | -0.016 | -0.627 | 0.272 | Illiterate | 0.439 |
| Chronic lung disease | -0.281 | -0.026 | -0.757 | 0.195 | Primary | 0.246 |
| Chronic lung disease | -0.185 | -0.014 | -0.723 | 0.353 | Middle | 0.501 |
| Chronic lung disease | -0.215 | -0.016 | -0.997 | 0.568 | High | 0.590 |
| Digestive system disease | -0.008 | -0.001 | -0.302 | 0.286 | Illiterate | 0.957 |
| Digestive system disease | 0.034 | 0.004 | -0.290 | 0.359 | Primary | 0.835 |
| Digestive system disease | -0.196 | -0.022 | -0.532 | 0.139 | Middle | 0.251 |
| Digestive system disease | -0.187 | -0.021 | -0.670 | 0.295 | High | 0.446 |
| Dyslipidaemia | 0.129 | 0.012 | -0.316 | 0.574 | Illiterate | 0.569 |
| Dyslipidaemia | 0.385 | 0.032 | -0.110 | 0.880 | Primary | 0.127 |
| Dyslipidaemia | 0.111 | 0.010 | -0.319 | 0.542 | Middle | 0.612 |
| Dyslipidaemia | -0.193 | -0.020 | -0.708 | 0.322 | High | 0.462 |
| Emotional and mental disorders | -0.587 | -0.016 | -1.931 | 0.758 | Illiterate | 0.392 |
| Emotional and mental disorders | -0.631 | -0.018 | -2.037 | 0.774 | Primary | 0.378 |
| Emotional and mental disorders | -1.664 | -0.039 | -3.269 | -0.059 | Middle | 0.042* |
| Emotional and mental disorders | -1.950 | -0.039 | -4.611 | 0.712 | High | 0.151 |
| Heart disease | 0.357 | 0.035 | -0.055 | 0.769 | Illiterate | 0.089 |
| Heart disease | 0.289 | 0.028 | -0.132 | 0.711 | Primary | 0.179 |
| Heart disease | 0.901 | 0.079 | 0.449 | 1.353 | Middle | <0.001*** |
| Heart disease | 0.138 | 0.014 | -0.421 | 0.697 | High | 0.628 |
| Hyperglycaemia | 0.011 | 0.001 | -0.513 | 0.535 | Illiterate | 0.967 |
| Hyperglycaemia | -0.399 | -0.027 | -0.994 | 0.197 | Primary | 0.189 |
| Hyperglycaemia | -0.265 | -0.018 | -0.822 | 0.292 | Middle | 0.351 |
| Hyperglycaemia | 0.120 | 0.009 | -0.596 | 0.837 | High | 0.742 |
| Hypertension | -0.166 | -0.022 | -0.464 | 0.133 | Illiterate | 0.277 |
| Hypertension | 0.239 | 0.030 | -0.093 | 0.571 | Primary | 0.159 |
| Hypertension | -0.106 | -0.013 | -0.443 | 0.230 | Middle | 0.536 |
| Hypertension | 0.133 | 0.017 | -0.302 | 0.568 | High | 0.550 |
| Kidney disease | 0.103 | 0.007 | -0.422 | 0.628 | Illiterate | 0.700 |
| Kidney disease | 0.064 | 0.004 | -0.525 | 0.653 | Primary | 0.832 |
| Kidney disease | 0.176 | 0.011 | -0.426 | 0.778 | Middle | 0.567 |
| Kidney disease | -0.141 | -0.009 | -0.925 | 0.644 | High | 0.725 |
| Liver disease | 0.162 | 0.008 | -0.562 | 0.886 | Illiterate | 0.661 |
| Liver disease | 0.738 | 0.040 | 0.011 | 1.464 | Primary | 0.047* |
| Liver disease | 0.258 | 0.014 | -0.448 | 0.963 | Middle | 0.474 |
| Liver disease | 0.343 | 0.019 | -0.575 | 1.260 | High | 0.464 |
| Memory related disease | -0.605 | -0.016 | -1.981 | 0.772 | Illiterate | 0.389 |
| Memory related disease | -0.911 | -0.024 | -2.390 | 0.567 | Primary | 0.227 |
| Memory related disease | -1.424 | -0.035 | -2.949 | 0.100 | Middle | 0.067 |
| Memory related disease | -2.325 | -0.070 | -4.114 | -0.537 | High | 0.011* |
| Stroke | 0.221 | 0.009 | -0.715 | 1.156 | Illiterate | 0.644 |
| Stroke | -1.382 | -0.056 | -2.350 | -0.415 | Primary | 0.005** |
| Stroke | -0.745 | -0.024 | -1.899 | 0.409 | Middle | 0.206 |
| Stroke | 0.632 | 0.020 | -1.031 | 2.295 | High | 0.456 |
| Troubled with body pain | -0.475 | -0.069 | -0.748 | -0.203 | Illiterate | 0.001** |
| Troubled with body pain | -0.601 | -0.082 | -0.907 | -0.295 | Primary | <0.001*** |
| Troubled with body pain | -0.287 | -0.035 | -0.608 | 0.034 | Middle | 0.080 |
| Troubled with body pain | -0.164 | -0.018 | -0.659 | 0.331 | High | 0.516 |
| Alcohol | 0.021 | 0.003 | -0.251 | 0.294 | Illiterate | 0.879 |
| Alcohol | 0.237 | 0.035 | -0.039 | 0.512 | Primary | 0.092 |
| Alcohol | 0.042 | 0.006 | -0.224 | 0.308 | Middle | 0.756 |
| Alcohol | 0.096 | 0.014 | -0.268 | 0.459 | High | 0.606 |
| Daily Sleep Time | -0.183 | -0.026 | -0.442 | 0.077 | Illiterate | 0.167 |
| Daily Sleep Time | -0.057 | -0.008 | -0.336 | 0.221 | Primary | 0.688 |
| Daily Sleep Time | -0.037 | -0.005 | -0.313 | 0.240 | Middle | 0.795 |
| Daily Sleep Time | -0.216 | -0.028 | -0.617 | 0.185 | High | 0.291 |
| Internet Use | 1.699 | 0.040 | 0.123 | 3.275 | Illiterate | 0.035* |
| Internet Use | 0.992 | 0.055 | 0.284 | 1.700 | Primary | 0.006** |
| Internet Use | 1.354 | 0.129 | 0.946 | 1.762 | Middle | <0.001*** |
| Internet Use | 1.151 | 0.151 | 0.724 | 1.578 | High | <0.001*** |
| Nap | 0.175 | 0.027 | -0.064 | 0.413 | Illiterate | 0.152 |
| Nap | 0.280 | 0.042 | 0.021 | 0.538 | Primary | 0.034* |
| Nap | 0.267 | 0.039 | 0.013 | 0.520 | Middle | 0.039* |
| Nap | -0.032 | -0.005 | -0.392 | 0.327 | High | 0.861 |
| Smoking | 0.059 | 0.009 | -0.219 | 0.337 | Illiterate | 0.676 |
| Smoking | -0.545 | -0.081 | -0.832 | -0.258 | Primary | <0.001*** |
| Smoking | -0.522 | -0.076 | -0.799 | -0.245 | Middle | <0.001*** |
| Smoking | -0.689 | -0.100 | -1.071 | -0.306 | High | <0.001*** |
| Social Activities | 0.086 | 0.013 | -0.152 | 0.324 | Illiterate | 0.479 |
| Social Activities | 0.219 | 0.033 | -0.042 | 0.480 | Primary | 0.100 |
| Social Activities | 0.535 | 0.076 | 0.273 | 0.797 | Middle | <0.001*** |
| Social Activities | 0.261 | 0.034 | -0.142 | 0.664 | High | 0.205 |
| Residential Area | 0.507 | 0.063 | 0.207 | 0.807 | Illiterate | 0.001** |
| Residential Area | 0.432 | 0.056 | 0.132 | 0.731 | Primary | 0.005** |
| Residential Area | 0.543 | 0.076 | 0.267 | 0.818 | Middle | <0.001*** |
| Residential Area | 0.926 | 0.136 | 0.551 | 1.300 | High | <0.001*** |
| Life Satisfaction | -0.268 | -0.022 | -0.712 | 0.176 | Illiterate | 0.237 |
| Life Satisfaction | -0.277 | -0.022 | -0.774 | 0.219 | Primary | 0.274 |
| Life Satisfaction | -0.068 | -0.005 | -0.586 | 0.450 | Middle | 0.796 |
| Life Satisfaction | -0.188 | -0.013 | -0.922 | 0.547 | High | 0.616 |
| (Intercept) | 21.038 | 21.038 | 17.842 | 24.234 | Illiterate | <0.001*** |
| (Intercept) | 18.723 | 18.723 | 15.649 | 21.798 | Primary | <0.001*** |
| (Intercept) | 17.305 | 17.305 | 13.923 | 20.687 | Middle | <0.001*** |
| (Intercept) | 21.106 | 21.106 | 16.699 | 25.513 | High | <0.001*** |
| *Note:* Group Illiterate: Illiterate; Group Primary: Primary school; Group Middle: Middle school; Group High: High school/vocational high school + Junior college or above. Significance levels: *P < 0.05, **P < 0.01, ***P < 0.001. | | | | | | |
