# Supplementary material for: Analysis of disparate factors affecting cognitive function among populations with different educational levels: a large-scale longitudinal study
Source: Front Psychol. 2026 Mar 19;17:1564721. doi: 10.3389/fpsyg.2026.1564721 (PMC13043331; doi:10.3389/fpsyg.2026.1564721)
Supplement: Supplementary file 2 [file table_2.docx]

| eTable 2. Mixed effects models of cognitive function across Three-Level educational stratification | | | | | | | | | | | | |
| --- | --- | --- | --- | --- | --- | --- | --- | --- | --- | --- | --- | --- |
|  | **Group Illiterate** | | | | **Group Primary + Middle** | | | | **Group High** | | | |
| **Variable** | **Model 1** | **Model 2** | **Model 3** | **Model 4** | **Model 1** | **Model 2** | **Model 3** | **Model 4** | **Model 1** | **Model 2** | **Model 3** | **Model 4** |
| (Intercept) | 15.888*** | 15.667*** | 15.677*** | 15.770*** | 18.096*** | 18.022*** | 18.003*** | 18.100*** | 20.251*** | 20.189*** | 20.035*** | 20.127*** |
| Age | -0.661*** | -0.668*** | -0.676*** | -0.665*** | -0.865*** | -0.823*** | -0.842*** | -0.824*** | -1.106*** | -1.024*** | -1.052*** | -1.033*** |
| Male | 0.148 | 0.127 | 0.118 | 0.125 | -0.248*** | -0.087 | -0.089 | -0.087 | -0.676*** | -0.449*** | -0.453*** | -0.450*** |
| Married | 0.049 | 0.006 | 0.016 | 0.012 | 0.108 | 0.063 | 0.066 | 0.068 | 0.153 | 0.079 | 0.064 | 0.054 |
| Smoking |  | 0.056 | 0.068 | 0.058 |  | -0.399*** | -0.384*** | -0.370*** |  | -0.668*** | -0.614*** | -0.607*** |
| Alcohol |  | 0.028 | 0.029 | 0.030 |  | 0.034 | 0.035 | 0.036 |  | -0.015 | -0.009 | -0.006 |
| Daily sleep time (≥ 8h) |  | -0.105 | -0.111 | -0.112 |  | -0.111 | -0.122* | -0.124* |  | -0.250* | -0.241* | -0.238* |
| Nap |  | 0.255** | 0.241** | 0.234** |  | 0.094 | 0.085 | 0.082 |  | 0.090 | 0.070 | 0.069 |
| Internet use |  | 0.299** | 0.259* | 0.254* |  | 0.322*** | 0.264*** | 0.260*** |  | 0.519*** | 0.416*** | 0.411*** |
| Social activities |  | 0.153* | 0.144 | 0.140 |  | 0.184*** | 0.181*** | 0.173*** |  | 0.185 | 0.177 | 0.179 |
| Residential area (Urban) |  |  | 0.192* | 0.188* |  |  | 0.255*** | 0.238*** |  |  | 0.440*** | 0.439*** |
| Life satisfaction (Dissatisfied) |  |  | -0.472*** | -0.478*** |  |  | -0.657*** | -0.640*** |  |  | -0.571** | -0.552** |
| Troubled with body pain |  |  |  | -0.030 |  |  |  | -0.047 |  |  |  | -0.026 |
| Hypertension |  |  |  | -0.067 |  |  |  | -0.059 |  |  |  | 0.008 |
| Dyslipidaemia |  |  |  | 0.144 |  |  |  | 0.320** |  |  |  | -0.093 |
| Hyperglycaemia |  |  |  | -0.367 |  |  |  | -0.271 |  |  |  | -0.143 |
| Cancer |  |  |  | 0.173 |  |  |  | -0.565 |  |  |  | 0.357 |
| Chronic lung disease |  |  |  | -0.318 |  |  |  | -0.335* |  |  |  | -0.471 |
| Liver disease |  |  |  | 0.113* |  |  |  | 0.103** |  |  |  | 0.041 |
| Heart disease |  |  |  | 0.357* |  |  |  | 0.545*** |  |  |  | 0.173 |
| Stroke |  |  |  | -0.086 |  |  |  | -0.742** |  |  |  | -0.435 |
| Kidney disease |  |  |  | 0.237 |  |  |  | -0.100 |  |  |  | 0.010 |
| Digestive system disease |  |  |  | -0.178 |  |  |  | -0.166 |  |  |  | -0.166 |
| Emotional and mental disorders |  |  |  | 0.003 |  |  |  | -0.941* |  |  |  | -0.501 |
| Memory related disease |  |  |  | -0.526 |  |  |  | -0.517 |  |  |  | -1.146 |
| Arthritis |  |  |  | -0.108 |  |  |  | -0.151 |  |  |  | -0.139 |
| Asthma |  |  |  | 0.146 |  |  |  | -0.226 |  |  |  | 0.895 |
| *Note:* Values represent standardized coefficients (* p < 0.05, ** p < 0.01, *** p < 0.001). Each model builds upon the previous one: The model presented in this table includes random intercepts for time and individual differences, Model 1: Demographics; Model 2: Model 1 + Lifestyle; Model 3: Model 2 + Environment; Model 4: Model 3 + Health Conditions. Group Illiterate: Illiterate; Group Primary: Primary school; Group Middle: Middle school; Group High: High school/vocational high school + Junior college or above. | | | | | | | | | | | | |
