# Supplementary material for: Analysis of disparate factors affecting cognitive function among populations with different educational levels: a large-scale longitudinal study
Source: Front Psychol. 2026 Mar 19;17:1564721. doi: 10.3389/fpsyg.2026.1564721 (PMC13043331; doi:10.3389/fpsyg.2026.1564721)
Supplement: Supplementary file 3 [file table_3.docx]

| eTable 3. Formal Interaction Tests: Educational Level as an Effect Modifier of Key Cognitive Function Determinants | | | | | |
| --- | --- | --- | --- | --- | --- |
| Predictor | Overall F-test | Pairwise Contrast | β (95% CI) | 95% CI | *P* value |
| Age | F(3) = 9.86; *P* < 0.001 *** | Primary vs. Illiterate | -0.022 | [-0.141, 0.098] | 0.723 |
|  |  | Junior High vs. Illiterate | -0.212** | [-0.338, -0.086] | 0.001 |
|  |  | High School+ vs. Illiterate | -0.337*** | [-0.484, -0.191] | < 0.001 |
| Sex (Male) | F(3) = 16.25; *P* < 0.001 *** | Primary vs. Illiterate | -0.357** | [-0.577, -0.137] | 0.001 |
|  |  | Junior High vs. Illiterate | -0.513*** | [-0.727, -0.299] | < 0.001 |
|  |  | High School+ vs. Illiterate | -0.86*** | [-1.110, -0.609] | < 0.001 |
| Internet Use | F(3) = 3.40; *P* = 0.017 * | Primary vs. Illiterate | -0.026 | [-0.287, 0.234] | 0.844 |
|  |  | Junior High vs. Illiterate | 0.159 | [-0.087, 0.405] | 0.204 |
|  |  | High School+ vs. Illiterate | 0.354* | [0.084, 0.624] | 0.010 |
| Urban Residence | F(3) = 1.52; *P* =0.208 | Primary vs. Illiterate | -0.001 | [-0.237, 0.234] | 0.991 |
|  |  | Junior High vs. Illiterate | 0.083 | [-0.141, 0.307] | 0.466 |
|  |  | High School+ vs. Illiterate | 0.239 | [-0.015, 0.492] | 0.065 |
| Memory-related Disease | F(3) = 1.20; *P* = 0.309 | Primary vs. Illiterate | 0.48 | [-0.946, 1.907] | 0.509 |
|  |  | Junior High vs. Illiterate | -0.711 | [-2.124, 0.703] | 0.324 |
|  |  | High School+ vs. Illiterate | -0.743 | [-2.246, 0.760] | 0.333 |
| *Note:* Shaded rows: overall Type III F-test for the interaction block (Satterthwaite df approximation); the F-statistic tests whether the predictor's effect on cognitive function differs significantly across all four educational groups as a whole. White rows: pairwise contrasts vs. Illiterate reference group derived from the same pooled mixed-effects model. β: unstandardized interaction coefficient (positive = predictor effect stronger in higher-education group; negative = weaker or reversed). Age was z-score standardized; all other predictors treated as factors. 95% CI: 95% confidence interval of the pairwise interaction coefficient. Not applicable (—) for F-test rows. Model: Pooled mixed-effects model. Fixed effects: five key predictors (age, sex, internet use, urban residence, memory-related disease) each interacted with educational level (× edu_factor); all remaining Model 4 covariates retained as main effects only. Random effects: (1\|ID) + (1\|year). Reference group: Illiterate. * p < 0.05; ** p < 0.01; *** p < 0.001 | | | | | |
